# Optimising pain management in children with acute otitis media through a primary care-based multifaceted educational intervention: study protocol for a cluster randomised controlled trial

**DOI:** 10.1186/s13063-018-2880-4

**Published:** 2018-09-17

**Authors:** Rick T. van Uum, Roderick P. Venekamp, Alies Sjoukes, Alma C. van de Pol, G. Ardine de Wit, Anne G. M. Schilder, Roger A. M. J. Damoiseaux

**Affiliations:** 1 Julius Center for Health Sciences and Primary Care, University Medical Center Utrecht, Utrecht University, Stratenum 5.143, PO Box 85500, 3508 GA Utrecht, The Netherlands; 2 Centre for Nutrition, Prevention and Healthcare, National Institute of Public Health and the Environment, Bilthoven, The Netherlands; 3 evidENT, Ear Institute, University College London, London, UK

**Keywords:** Acute otitis media, Pain management, Analgesics, Multifaceted intervention, Primary care, RCT

## Abstract

**Background:**

Whilst current guidelines highlight the importance of pain management for children with acute otitis media (AOM), there is evidence to suggest that this is not implemented in everyday practice. We have developed a primary care-based multifaceted educational intervention to optimise pain management in children with AOM, and we trial its clinical and cost effectiveness.

**Methods:**

This cluster randomised controlled trial aims to recruit 250 children aged 6 months to 10 years presenting with AOM to general practitioners (GPs) in 30 primary care centres (PCCs) across the Netherlands. GPs in the PCCs allocated to the intervention group receive a blended GP educational programme (online and face-to-face training). The intervention asks GPs to proactively discuss pain management with parents using an information leaflet, and to prescribe paracetamol and ibuprofen according to current guidelines. GPs in both groups complete an online module illustrating various otoscopic images to standardise AOM diagnosis. GPs in the PCCs allocated to the control group do not receive any further training and provide ‘care as usual’.

During the 4-week follow-up, parents complete a symptom diary. The primary outcome is the difference in parent-reported mean earache scores over the first 3 days. Secondary outcomes include both number of days with earache and fever, GP re-consultations for AOM, antibiotic prescriptions, and costs. Analysis will be by intention-to-treat.

**Discussion:**

The optimal use of analgesics through the multifaceted intervention may provide symptom relief and thereby reduce re-consultations and antibiotic prescriptions in children with AOM.

**Trial registration:**

Netherlands Trial Register, NTR4920. Registered on 19 December 2014.

**Electronic supplementary material:**

The online version of this article (10.1186/s13063-018-2880-4) contains supplementary material, which is available to authorized users.

## Background

Analgesics are the cornerstone of childhood acute otitis media (AOM) management. Optimal use of analgesics provides symptom relief and has the potential to reduce re-consultations, antibiotic prescription, and healthcare costs. Current AOM practice guidelines therefore emphasise the importance of providing analgesics to all children with AOM in an weight-appropriate dose, in addition to prescribing antibiotics for certain children [1, 2].

In daily practice, little attention is paid to earache management during the medical consultation [3–6] and routine antibiotic prescription is still very common [3, 7, 8]. Key factors driving the management decisions of general practitioners (GPs) include concerns from both clinicians and parents about the vulnerability of young children and parental pressure to prescribe antibiotics; parents believe that antibiotics are the proper treatment for AOM and analgesics as a standalone treatment are generally considered insufficient [9–11].

Multifaceted interventions addressing these concerns from both the GP and parent perspective have proven effective in changing clinical practice [12, 13] and are highly valued by GPs [14]. We therefore developed a primary care-based multifaceted educational intervention to optimise pain management in children with AOM and we will trial its clinical and cost effectiveness.

## Objective

We aim to establish the clinical and cost effectiveness of a primary care-based multifaceted educational intervention focused on optimising pain management in children with AOM.

## Methods and analysis

### Design of the intervention to optimise pain management in children with AOM

The Medical Research Council guidance for complex interventions was used to develop our multifaceted intervention [15]. The various elements of the intervention were chosen based on a literature review, consultation with clinical experts including a broad range of disciplines such as GPs, an ear, nose, and throat (ENT) surgeon, and a nurse specialist as well as educational experts, and a qualitative pre-study with GPs and parents.

Following this multi-phase process, we found that multifaceted interventions including a GP educational programme with condensed information of a clinical guideline may impact GP prescribing [12, 13, 16, 17], and allows for addressing parental factors influencing the management decisions of GPs [6, 11]. Individual online training appeared to be more effective than face-to-face group work [18], and the impact may increase when online training is complemented by individual face-to-face visits to convey feedback on performance in the training and to overcome obstacles to change [19]. In addition, patient information leaflets have the potential to impact GP prescribing and guide parental management decisions [20, 21].

Our multifaceted intervention therefore comprises of four core elements to educate GPs in different aspects (knowledge, attitude, skills, and behaviour): i) an online training module; ii) a face-to-face-meeting; iii) a parent information leaflet; and iv) prescription of analgesics (see Table 1).

**Table 1:** Main topics in the blended learning module

Topic	Intervention element	Educational component	Educational level
Otoscopy, including abnormal tympanic membrane appearances (AOM, OME)	Online training module (both intervention and control)	Case-based learning Self-assessment with immediate feedback (MC)	Knowledge Skills
Prevalence and natural course of earache due to AOM	Online training module	Case-based learning Self-assessment with immediate feedback (MC)	Knowledge
(Limited) effect of antibiotics on AOM symptoms, including earache	Online training module	Case-based learning Self-assessment with immediate feedback (MC)	Knowledge
Safety and adverse effects of paracetamol and ibuprofen	Online training module	Case-based learning Self-assessment with immediate feedback (MC)	Knowledge
Parents’ beliefs, concerns, and expectations relating to (earache due to) AOM and analgesics use	Online training module Face-to-face training	Case-based learning Open questions	Knowledge
GP barriers to prescribe paracetamol and/or ibuprofen	Online training module Face-to-face training	Open questions	Knowledge Attitude
Dosing and timing of analgesics according to the 2007 “Pain Relief” guideline [24] (and subsequent 2014 “AOM in children” guideline [1])	Online training module Face-to-face training Information leaflet	Case-based learning Self-assessment with immediate feedback (MC)	Knowledge Skills
How to use effective communication skills to address pain management in a consultation	Online training module	Video demonstration	Skills
Use the three communication elements of an effective consultation [13, 21]: • explore beliefs, concerns and expectations of parents regarding the use of analgesia • stress the importance of analgesic treatment during the consultations and agree with the parents on pain management • check parents’ understanding	Online training module	Video demonstration	Behaviour Attitude
Use a parent information leaflet during the consultation	Face-to-face training Information leaflet	Lecture/presentation	Behaviour Attitude
Prescribe paracetamol and ibuprofen	Face-to-face training Information leaflet	Lecture/presentation	Behaviour Attitude

*AOM* acute otitis media, *GP* general practitioner, *MC* multiple choice, *OME* otitis media with effusion

#### Online training module

The online training module, which takes approximately 30 min to complete, educates GPs about pain management in childhood AOM through a combination of proven effective educational components such as case-based learning [22], self-assessment with immediate feedback [22], reflection [22], and video demonstrations of effective communication techniques [13, 23]. GPs are trained to proactively discuss pain management with parents using the parent information leaflet and they are prompted to prescribe both paracetamol and ibuprofen according to current AOM guidelines [1, 24].

GP adherence to the online training module (i.e. whether the GP completed the module), as well as individual answers by GPs to the various questions, will be automatically recorded in the digital Julius Center CME platform. The full content of the module will be made available at the trial website (www.pimpomstudie.nl) after completion of the trial.

#### Face-to-face meeting

Upon completion of the online training module by GPs in the intervention primary care centre (PCC), a face-to-face meeting with the co-ordinating investigator and the GPs will be scheduled at the GP’s PCC to ensure engagement. This also provides an opportunity to discuss the main topics of the online training module (see above and Table 1) and potential barriers and facilitators to analgesic prescription.

#### Parent information leaflet

The parent information leaflet is illustrated in Additional file 1; it explains the importance of adequate pain management and includes tables of weight-appropriate dosing of paracetamol and ibuprofen based on prevailing Dutch guidelines [1, 24]. The leaflet also debunks common myths and misconceptions about the use of analgesics in children [10, 11].

#### Prescription of analgesics

Despite paracetamol and ibuprofen being available over-the-counter in the Netherlands, GPs in the intervention group are requested to prescribe these drugs at a weight-appropriate dose [1, 24] and request parents to fill these prescriptions at the local pharmacy the same day.

### Randomised controlled trial of the intervention to optimise pain management in children with AOM

#### Study design and setting

**Fig. 1:**
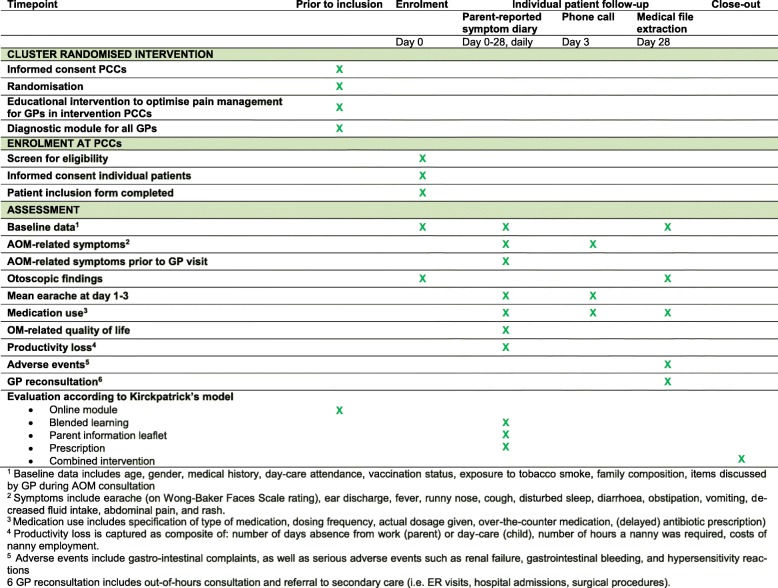
Overview and schedule of enrolment, data collection, and assessments (SPIRIT Figure). ^1^Baseline data includes age, gender, medical history, day-care attendance, vaccination status, exposure to tobacco smoke, family composition, and items discussed by general practitioner (GP) during acute otitis media (AOM) consultation. ^2^Symptoms include earache (on Wong-Baker Faces Scale rating), ear discharge, fever, runny nose, cough, disturbed sleep, diarrhoea, obstipation, vomiting, decreased fluid intake, abdominal pain, and rash. ^3^Medication use includes specification of type of medication, dosing frequency, actual dosage given, over-the-counter medication, (delayed) antibiotic prescription. ^4^Productivity loss is captured as composite of number of days absence from work (parent) or day-care (child), number of hours a nanny was required, and costs of nanny employment. ^5^Adverse events include gastrointestinal complaints, as well as serious adverse events such as renal failure, gastrointestinal bleeding, and hypersensitivity reactions. ^6^GP re-consultation includes out-of-hours consultation and referral to secondary care (i.e. emergency room visits, hospital admissions, and surgical procedures). PCC primary care centre

We designed a pragmatic, cluster randomised controlled trial to assess the clinical and cost effectiveness of the primary-care based multifaceted educational intervention aimed at optimising pain management compared with ‘care as usual’ in children with AOM. A SPIRIT checklist is attached as Additional file 2, and the SPIRIT figure (Fig. 1) shows the study design.

Approximately 30 general practices in multiple regions across the Netherlands will participate and enrol children to the trial. The trial recruitment period is 3 years with a follow-up of individuals for 4 weeks. Participating PCCs will be revisited every year in the autumn (prior to the annual peak incidence of AOM) to refresh key elements of the intervention and study procedures.

#### Randomisation and blinding

The unit of randomisation is the PCC; participating centres are randomly allocated to either the multifaceted education intervention or the usual care group. GPs in the same PCC will thus be allocated to the same ‘treatment’ group to avoid contamination issues.

A trial website has been designed to randomly assign participating PCCs to either the intervention or the control group. To ensure equal distribution of PCC characteristics (size and age distribution) across ‘treatment’ groups, an independent statistician has designed a computerised minimisation strategy with a random component of 30% [25].

We will perform an open-label trial, in other words no blinding will be performed. However, to avoid contamination between intervention and control PCCs during enrolment as much as possible, GPs in the control group are asked to participate in a study to monitor earache in children with AOM.

To ensure standardised AOM diagnosis, each participating GP receives unique user credentials to access the digital Julius Center CME platform containing an online module illustrating various otoscopic images (i.e. normal appearance of tympanic membrane, AOM, and otitis media with effusion). GPs in the intervention PCCs receive additional access to the online training module.

#### Eligibility criteria

Children aged 6 months to 10 years presenting to their GP with earache and AOM are eligible for inclusion. Those with ventilation tubes in place are excluded, as well as previously included children, those with a previously included sibling, and children with either Down’s syndrome, craniofacial malformations, known immunodeficiencies, liver failure, or renal insufficiency.

#### Inclusion and baseline assessments

All participating GPs inform the parents of potentially eligible children about the trial. After obtaining informed consent, GPs complete a short questionnaire regarding the child’s medical history (recurrent AOM, recurrent upper respiratory tract infection, previous ENT surgery, and atopy) and perform a short physical examination (presence of fever and appearance of the tympanic membrane).

Participating GPs are also asked to complete non-recruitment logs, documenting reasons for non-recruitment of eligible patients and eligible patients who declined to participate.

GPs in the intervention group will advise parents of children on analgesics according to the instructions of the multifaceted intervention (see above). Other clinical decisions such as antibiotic prescriptions are at the discretion of the GP. Children in the control group will receive ‘care as usual’ and complete the same study procedures as those in the intervention group.

#### Follow-up data collection

At inclusion, GPs provide parents with a study diary to report various outcomes (see Fig. 1). Parents complete a daily symptom diary for 2 consecutive weeks. At baseline, and at 2 and 4 weeks, parents complete quality of life questionnaires and, at 4 weeks, parents fill out a productivity loss questionnaire. The study team will contact parents by phone on day 3 to optimise parents’ compliance and to capture critical data on our primary outcome (Fig. 1). One month after inclusion, the co-ordinating investigator will contact parents by telephone or email with a reminder to return the completed diary by mail.

After 4 weeks, the coordinating investigator will visit the PCC to retrieve the data of participating children from their medical records (Fig. 1).

#### Validated questionnaires used

Parents report their child’s earache intensity using the Wong-Baker FACES® Pain Rating Scale (scores range from 0 to 10, with lower scores indicating less pain) [26–29]. Disease-specific quality of life of the child is assessed at baseline and at 4 weeks with the parent-reported Otitis Media-6 (OM-6), a six-item questionnaire recording ear-related problems (scores range from 6 to 42, with lower scores indicating better quality of life) [30]. Quality of life of the parents is assessed at baseline and at 2 weeks with the EuroQOL five dimensions quality of life questionnaire (EQ-5D) [31], and productivity losses are assessed with an adapted version of the iMTA Productivity Cost Questionnaire (iPCQ) [32].

#### Primary and secondary outcomes

The primary outcome of interest is the difference in parent-reported mean earache score over the first 3 days. Secondary outcomes are the number of days with earache and earache severity, number of days with fever, the proportion of children with earache at various time points (24 h, 2 to 3 days, 4 to 7 days), GP re-consultation because of AOM, antibiotic prescriptions because of AOM, health-related quality of life (HRQoL) of the child and their parents, working days lost for the parents, days lost from day-care or school for children, complications of AOM, (serious) adverse events of analgesics, healthcare use, and cost effectiveness.

#### Sample size calculation

In a previous childhood AOM trial, the mean earache score on days 1 to 3 was 3.7 (standard deviation 2.57) [33]. We consider a 25% reduction of this mean earache score clinically relevant. With 80% power, at a 5% significance level, a minimum of 66 children per group is needed. The inflation factor for the cluster design is 1.7 assuming an intra-class correlation coefficient of 0.05 (for PCC level) [34, 35] and a cluster size of 15 children (anticipating that not all PCCs recruit children to the trial). As such, the number of children that need to be included per group will be 115. Based on previous experience, we consider a loss to follow-up of 10% to be reasonable [36, 37], and therefore aim to include 125 children per group (250 children in total).

#### Statistical analysis

Analysis will be performed according to the intention-to-treat principle.

##### Clinical effectiveness

For our primary outcome, we will calculate the effect of the multifaceted intervention on parent-reported mean earache score over the first 3 days using a linear mixed model. A random intercept for PCC will be included in the model to account for cluster randomisation and a residual covariance (i.e. generalised estimating equation type) matrix will be included for repeated measurements (days 1, 2, and 3 after the initial GP visit). Clustering is estimated with an intra-class correlation. Treatment effects will be reported as the crude and adjusted difference in mean earache scores over the first 3 days between study groups with accompanying 95% confidence intervals. We plan to adjust for baseline differences in pre-specified confounders [38], including age, gender, ill appearance, fever, history of recurrent AOM, antibiotic use, and day-care attendance. Missing data at baseline will be imputed using the multivariate imputation by chained equations (MICE) procedure [39]. The imputed data sets will be analysed, and results combined. We will average estimates of the treatment effects to give a single mean estimate and adjusted standard errors according to Rubin’s rule [40]. In primary analysis, we will include all patients for whom the outcome was observed. In a sensitivity analysis, or in case of extensive missing data, we will impute missing outcome data using the multiple imputation techniques described above [41]. Where appropriate, we will also perform an adherence-adjusted analysis.

For our secondary outcomes, we will use mixed logistic regression analyses for dichotomous variables, and mixed Poisson regression analyses for count variables. In case of insufficient or non-events, for example very few re-consultations, we will perform a simplified analysis without adjustment for confounders and clustering.

##### Cost-effectiveness analysis (CEA)

We will take medical and non-medical direct and indirect costs into account, thus using a societal perspective for this analysis. All analyses will use a time horizon of 4 weeks, corresponding to the follow-up period in the study. Therefore, discounting is not applicable.

We will use a Dutch database of current drug prices (www.medicijnkosten.nl) to estimate costs of patient drug use. These costs will be increased with a pharmacist’s charge. We will base the costs of over-the-counter medication and complementary medication, if used, on average retail prices. We will base the costs of consulting a GP or a medical specialist, or other procedures and hospitalisations, on current Dutch guidelines for pharmaco-economic evaluation [42]. These guidelines include reference cost figures for use in health economic evaluations for common types of healthcare use. We extract resource use from the patient diaries. This includes doctor visits, prescribed medication including antibiotics, specialist referrals, hospital admissions, and surgical interventions, as well as out-of-pocket expenses such as over-the-counter medication, child care, and travel costs. When no reference prices are available, we will calculate cost prices according to guidelines for economic evaluation in healthcare research [42]. Indirect costs to society associated with absence from work will be estimated using the friction cost method [43]. Overall costs will be compared across the study groups and, where relevant, we will calculate differences, including 95% confidence intervals, using two-stage non-parametric bootstrap sampling (of clusters and participants) [44].

In cost-effectiveness analysis, we will calculate incremental cost-effectiveness ratios by dividing the estimated differences in costs by the differences in effects observed, i.e. the additional cost per point reduction in mean earache score on days 1 to 3, for the optimised pain management strategy compared with the ‘usual care’ strategy.

We will address uncertainty by means of two-stage non-parametric bootstrap sampling (of clusters and participants) [44]. We will use net benefit regression methods to study the effect of any differences in baseline characteristics and cluster differences on cost-effectiveness results. We will compare the results of the two-stage bootstrap sampling and the multilevel net benefit regression methods to assess robustness of the cost-effectiveness estimates. We will present the final results using incremental cost-effectiveness planes and cost-effectiveness acceptability curves.

#### Process evaluation: understanding change processes

During the recruitment phase, we will perform two qualitative studies as process evaluations alongside our trial to unravel mechanisms by which the multifaceted intervention may sort its effects [45–47]. In both studies we will use semi-structured interviews, which are audio-recorded and transcribed verbatim. Interviews will be analysed thematically using a grounded theory approach by all members of the multidisciplinary research team, including a primary healthcare sociologist, an ENT surgeon, an educational specialist, and academic GPs. We expect to reach saturation after 10–20 interviews in each study based on previous research [11, 48].

In one qualitative study, we will interview a subset of GPs in the intervention PCCs using purposeful sampling. In these interviews, the views and expectations of GPs on analgesia and how the multifaceted educational intervention shaped these perceptions will be explored. In a second qualitative study, we will perform interviews with parents from both the intervention and control groups to investigate their perceptions towards earache, AOM, and its management (with a specific focus on analgesia), and their experiences with AOM-related doctor consultations in general and the baseline study visit in particular. The results of these process evaluations will facilitate the interpretation of trial results. We also plan to conduct a further evaluation according to Kirkpatrick’s evaluation model [49] (see Table 2) which may aid future implementation of the intervention.

**Table 2:** Evaluation of the intervention according to Kirkpatrick’s model

Intervention	Evaluation method	Evaluated aspect
Online module	Audio-recorded evaluation during the face-to-face visit with the coordinating investigator	Reaction/satisfaction
Blended learning	Questionnaire at baseline in the parental diary assessing whether analgesic treatment was explicitly discussed during the consultation	Knowledge/behaviour
Parent information leaflet	Questionnaire at baseline in the parental diary assessing whether the leaflet • has been discussed during the consultation • has been read by the parents and whether they found it useful	Knowledge/behaviour Reaction/satisfaction
Prescription	Questionnaire at baseline in the parental diary assessing whether the general practitioner prescribed paracetamol and/or ibuprofen	Knowledge/behaviour
Combined intervention	Audio-recorded evaluation during the close-out visit of the study	Reaction/satisfaction

### Implementation of the multifaceted intervention

The results of this research project will be disseminated through publications in peer-reviewed professional journals, and will be presented at national and international conferences. If our intervention proves effective, we will liaise with all relevant stakeholders including GPs, parents of children with AOM, and the Dutch College of General Practitioners (Nederlands Huisartsen Genootschap) to develop a dissemination and implementation plan to ensure rapid and effective dissemination and translation into clinical practice.

### Data management

#### Data monitoring

Although monitoring is not strictly necessary in the Netherlands for studies to which the Dutch Medical Research Involving Human Subjects Act (WMO) does not apply, we will have our study monitored yearly by an independent Data Monitoring Committee at the University Medical Center Utrecht, in the shape of a low-risk research data monitor, to ensure the quality of the trial execution.

#### Data deposition and curation

Each participant is assigned a unique study identification number. This enables us to handle and store data in a non-traceable manner within a secured and coded database designed by the data management department of the Julius Center. Paper-based data are automatically entered on an online database (Research Online) which our data management department develops and maintains. After termination of the study, the database will become available to authorised persons (investigators, monitors, auditors) only. We have not planned any interim analyses.

Upon completion of the trial, data are stored for the at least 15 years on a central drive of the data management department of the Julius Center and will be made available for the use by third parties upon request and approval of the principal investigator (RAMJD).

## Discussion

To our knowledge, this trial is unique in focusing on optimising analgesia to reduce pain and antibiotic use in childhood acute otitis media. The trial intervention has been developed systematically in close collaboration with educational and clinical experts and parents of children with AOM. The trial will assess both clinical and cost effectiveness, as well as underlying mechanisms through parallel process evaluations.

Even though the intervention is targeted at GPs, the impact is measured at the patient level which potentially underestimates the benefit of optimising pain management. However, we deem an individually randomised, placebo-controlled trial unethical since analgesics are widely accepted as standard care in children with AOM.

Findings from the trial and parallel process evaluations may contribute to optimisation of childhood AOM management.

### Trial status

The trial design has been registered prior to enrolment on 19 December 2014 in a public trial registry (Netherlands Trial Register, NTR4920). At the time of submission of this manuscript, 218 patients have been recruited into the trial.

## Additional file

Additional file 1: Information leaflet about pain relief for children with middle ear infection. (PDF 665 kb)

Additional file 2: SPIRIT 2013 checklist: recommended items to address in a clinical trial protocol and related documents. (DOC 120 kb)

## Abbreviations

AOM: Acute otitis media
ENT: Ear, nose, and throat
EQ-5D: EuroQol five dimensions quality of life questionnaire
GP: General practitioner
HRQoL: Health-related quality of life
iPCQ: iMTA Productivity Cost Questionnaire
OM-6: Otitis Media-6
PCC: Primary care centre
WMO: Dutch Medical Research Involving Human Subjects Act
